# Dementia is the second most feared condition among Australian health service consumers: results of a cross-sectional survey

**DOI:** 10.1186/s12889-023-15772-y

**Published:** 2023-05-12

**Authors:** Rochelle Watson, Robert Sanson-Fisher, Jamie Bryant, Elise Mansfield

**Affiliations:** 1 Health Behaviour Research Collaborative, School of Medicine and Public Health, College of Health, Medicine and Wellbeing, University of Newcastle, W4, HMRI Building, Callaghan, NSW 2308 Australia; 2 Equity in Health and Wellbeing Research Program, Hunter Medical Research Institute, New Lambton Heights, NSW Australia

**Keywords:** Dementia, Fear, Community awareness

## Abstract

Fear of disease may act as a barrier to screening or early diagnosis. This cross-sectional survey of 355 people attending outpatient clinics at one Australian hospital found that cancer (34%) and dementia (29%) were the most feared diseases. Participants aged 65 years and over feared dementia the most.

## Background

Dementia is a leading cause of death and disease burden in Australia [1]. In 2018, dementia accounted for 4% of the total burden, ranking 3rd in the top 20 causes of burden overall, and in the top 2 causes for people aged over 75 years [2]. In 2022 it is estimated there are 487,500 adults living with dementia in Australia [3]. The risk of developing dementia increases with age, with one in 10 Australians aged over 65 years and three in 10 people aged over 85 years diagnosed with dementia [4]. A timely diagnosis of dementia can have important benefits such as: enabling early and proactive intervention to sustain independence for longer; allowing for advance personal planning while the person with dementia may still have capacity; and providing more time to become educated about dementia and establish contact with support services and networks [5].

In line with models of Health Anxiety, it is likely that a subset of individuals who are concerned about a health condition may delay or avoid seeking medical advice when early signs and symptoms are noticed [6]. This has previously been reported in studies of cancer help seeking behaviour, where participants have reported fear of diagnosis as a key reason for not seeking medical attention [7]. Fear of dementia, or ‘Dementia Worry’ [8] may act as a barrier to early diagnosis. Although dementia worry is generally associated with a greater likelihood of seeking a diagnosis, more than 10% of people report that they would not agree to be screened if they detected issues with their memory or cognitive function, and 30% report hesitation in agreeing to be screened [9]. A recent review of barriers and facilitators to obtaining a dementia diagnosis found that dementia stigma and fear of a diagnosis were significant contributors to delayed help-seeking in eight studies [10].

In 2010–2011, a series of international studies conducted in the United Kingdom [11], United States [12], France [13], Germany [13], and Spain [13] found that Alzheimer’s disease, the most common form of dementia, [14] was the second most feared disease, closely following cancer. A study conducted in the United States that looked at changes in feared diseases between 2006 and 2011 found that the proportion of participants indicating Alzheimer’s as their most feared disease had the greatest increase over time than any of the other illnesses explored [12]. Research in Australia in 2010 found that 63% of people who participated in an online survey feared dementia, which was second only to cancer (66%) [15]. Another Australian study conducted in 2011 echoed these results, with 27% ranking cancer as their biggest fear or their future health, and 11.1% ranking dementia in the top position [16].

International studies have found conflicting results in relation to the impact of age and gender on fear of dementia. A Canadian study of 245 healthy adults found that younger participants self-reported a greater fear of dementia than older participants [17]. Conversely, a German study found that being female and older age were associated with greater fear of dementia among a large sample of the general population [18]. Differences associated with age and gender may be expected given the death and disease burden of dementia in Australia is greater for women than men, and increases with age. [2] However, such differences have not been reported in an Australian setting.

Previous research exploring the most feared diseases did not report on the reasons that people feared these conditions. This limits the potential to identify where community education may be targeted to alleviate fear and reduce potential delays in help-seeking. Substantial literature describes factors which contribute to fear of a cancer diagnosis, which include fears about possible symptoms, physical, social, emotional implications of cancer, and fears about mortality [11]. Several studies have also explored the factors that may contribute to an increased level to dementia worry, including personal experience with dementia, depression and poor subjective health [19–22]. In a 2017 study, participants reported the reasons they would be concerned to share a diagnosis of dementia with family and friends, primarily citing fear that people would treat them, and think about them differently [9]. This study aims to update data about feared diseases in an Australian setting and provide some insight into the general reasons people fear the top listed diseases.

## Methods

### Aims

This study aimed to explore among Australian health service consumers:

1. The most feared health conditions from the top ten causes of fatal burden in Australia [23];
2. Differences regarding the most feared health conditions by age and gender; and.
3. Differences in the main reasons for the top feared conditions.

### Design and setting

A cross-sectional survey of community members attending outpatient clinics at one large, regional Australian hospital was conducted. Outpatient clinics included orthopaedics, cardiology, renal, urology, and general surgery outpatient clinics. The study protocol was reviewed and approved by the Human Research Ethics Committee with oversight of the health district where the participating hospital was located (reference number: 17/03/15/4.06).

### Sample

Eligible participants were adults who were attending an outpatient clinic at the participating hospital; had sufficient English to participate; indicated they were physically well enough to complete an iPad survey; and provided verbal informed consent.

### Recruitment and data collection

Trained research assistants systematically approached potential participants in outpatient clinic waiting rooms and invited participation over a three-month period. Some patients were not invited to participate if they were called in to their appointment prior to being approached. Potential participants who indicated they were well enough to complete the survey, and provided verbal consent to participate, were asked to complete an anonymous online survey on a web-connected iPad. The age category (18–34, 35–44, 45–54, 55–64, 65–74, 75+) and gender of non-consenters was recorded with their permission to enable examination of consent bias.

### Measures

Participants self-reported their age (open text item) and gender (male, female), then were asked “Regardless of any health problems you have had, which of the following conditions do you most fear?”. Response options were drawn from the most recent data at the time of survey development regarding the leading causes of fatal burden (years lost due to premature death) in Australia [23]. Response options included: “Coronary heart disease (not high blood pressure or high cholesterol); Dementia and Alzheimer’s disease; Cerebrovascular disease (e.g. Stroke); Cancer; Chronic obstructive pulmonary disease (COPD) (e.g. Emphysema); Diabetes; Heart failure; None of these”. Participants were instructed to select one option only. To ascertain the reasons for their selection, participants were asked to indicate up to three most important reasons from the following list: “Shortened length of life; Emotional impact (e.g. depression); Social impact (e.g. stigma, feeling isolated); Practical issues (e.g. needing help to do daily tasks); Financial strain (e.g. unable to work or pay for treatments); Physical symptoms and side-effects (e.g. pain, fatigue, vomiting); Legal issues (e.g. appointing someone to make decisions on my behalf); Other”. Where participants selected “Other” they were able to input additional text explaining the reasons. Rather than specific disease-related reasons, the response options were intended to be general in nature, reflecting the various ways the listed diseases may impact on a person’s life. The survey was pilot tested with a sample of 20 participants to check acceptability and relevance of the items.

### Statistical analysis

Statistical analyses were conducted using IBM SPSS Statistics v23 [24]. Age and gender of consenters versus non-consenters were compared using chi-square statistics to examine consent bias. Descriptive statistics were calculated to examine the most feared health condition and reasons for fear. Chi-square statistics were calculated to explore differences regarding the most-feared health conditions by categories of age and gender and differences in the reasons selected between participants who chose cancer versus dementia as their most feared condition. Where cells had expected counts less than five, the Monte Carlo method (based on 10,000 sampled tables with starting seed 2,000,000) was used to provide an unbiased estimate of the exact p value as the data set was too large to calculate an exact p value. Sixty-five years was used as the cut-off between the younger and older age groups to correspond with the definition of older Australians used by the Australian Institute of Health and Welfare [25].

## Results

### Sample

Of 505 eligible participants approached, 355 (70.3%) consented to participate. There was no significant difference in gender between consenters and non-consenters (χ^2^ = 2.67, V = 0.07, p = 0.102). However, there was a significant difference in age (χ^2^ = 17.24, V = 0.22, p = 0.007), with a greater proportion of non-consenters in the 65–74 years age category than for consenters. There was an approximately even distribution of male (47%) and female (53%) participants, with a mean age of 55 years (SD = 16.9).

### Most feared health conditions

Responses by age, gender, and total persons regarding the most feared health conditions are presented in Table 1. Overall, the most feared condition was cancer (33.5%, 95%CI 28.5–38.3) followed by dementia (29.3%, 95%CI 24.5–34.4), and heart failure (8.5%, 95%CI 5.9–11.5). There was no significant difference in the most feared condition by gender (χ^2^ = 5.55, V = 0.13, p = 0.697). However, there was a significant difference in the most feared condition between younger and older participants (χ^2^ = 17.24, V = 0.22, p = 0.022, 99%CI for p 0.018–0.025 by Monte Carlo method). While cancer and dementia remained the top two most feared conditions for both age groups, among younger participants (aged 18–64 years) cancer was the most feared condition (38.1%, 95%CI 32-44.3), while among those aged 65 years and over dementia was the most feared (32.8%, 95%CI 24.6–41.9).

**Table 1:** Most feared conditions by age, gender and total persons

Health Condition	Age (Years)		Gender		Total Persons (n = 355)
	18–64 (n = 236)	65+ (n = 119)	Male (n = 166)	Female (n = 189)	
Coronary heart disease	11 (4.7%)	15 (12.6%)	14 (8.4%)	12 (6.3%)	26 (7.3%)
Dementia / Alzheimer’s disease	65 (27.5%)	39 (32.8%)	44 (26.5%)	60 (31.7%)	104 (29.3%)
Cerebrovascular disease	7 (3%)	6 (5%)	6 (3.6%)	7 (3.7%)	13 (3.7%)
Cancer	90 (38.1%)	29 (24.4%)	57 (34.3%)	62 (32.8%)	119 (33.5%)
Chronic obstructive pulmonary disease	10 (4.2%)	2 (1.7%)	4 (2.4%)	8 (4.2%)	12 (3.4%)
Diabetes	13 (5.5%)	11 (9.2%)	14 (8.4%)	10 (5.3%)	24 (6.8%)
Heart failure	22 (9.3%)	8 (6.7%)	16 (9.6%)	14 (7.4%)	30 (8.5%)
Other	4 (1.7%)	1 (0.8%)	1 (0.6%)	4 (2.1%)	5 (1.4%)
None of these	14 (5.9%)	8 (6.7%)	10 (6%)	12 (6.3%)	22 (6.2%)

### Reasons for the most feared conditions

Reasons for fear were only explored for participants who selected dementia (n = 103), cancer (n = 111) and heart failure (n = 30) as their most feared condition as these accounted for the largest proportion of responses (Table 2). Among the ‘other’ reasons provided by participants for fearing the top conditions, family history was cited most frequently (dementia n = 4, cancer n = 9, heart failure n = 1).

**Table 2:** Reasons for fear of conditions

Reason	Dementia (n = 104)		Cancer (n = 119)		Heart Failure (n = 30)	
	*n*	*%*	*n*	*%*	*n*	*%*
Emotional impact	62	60.2	51	42.9	6	20
Practical issues	61	59.2	32	26.9	10	33.3
Social impact	41	39.8	6	5	3	10
Physical symptoms and side effects	25	24.3	68	57.1	14	46.7
Shortened length of life	16	15.5	82	68.9	16	53.3
Legal issues	14	13.6	5	4.2	-	-
Financial strain	10	9.7	32	26.9	3	10
Other	11	10.7	16	13.4	4	13.3

### Differences in reasons between participants who feared cancer versus dementia

There were statistically significant differences in the reasons for fear of cancer versus fear of dementia. Significantly more participants who selected cancer as their most feared condition than who selected dementia reported shortened length of life (χ^2^ = 63.79, V = 0.54, p < 0.001), physical symptoms and side effects (χ^2^ = 24.51, V = 0.33, p < 0.001), and financial strain (χ^2^ = 10.63, V = 0.22, p = 0.001) in their top three reasons. Significantly more participants who selected dementia, as compared to those who selected cancer, reported social impact (χ^2^ = 39.98, V = 0.42, p < 0.001), practical issues (χ^2^ = 23.71, V = 0.33, p < 0.001), emotional impact (χ^2^ = 6.64, V = 0.17, p = 0.01), and legal issues (χ^2^ = 6.22, V = 0.17, p = 0.013) as top reasons for fear. There was no significant difference in the proportion of participants reporting family history as a reason between those who most feared cancer versus dementia (χ^2^ = 1.40, V = 0.08, p = 0.237).

## Discussion

Cancer and dementia were selected by the majority of participants as the most feared conditions, followed by heart failure. Interestingly, cancer and dementia outranked coronary heart disease, the fourth most feared condition, despite this being the leading cause of fatal burden in Australia [2]. Deteriorating health and wellbeing [7, 26, 27], intensive [7] or limited treatment options [28], and negative impact on loved ones [29–31] are commonly associated with both cancer and dementia. It is possible that the impact of cancer and dementia may be considered more threatening than a heart attack or angina resulting from coronary heart disease.

The finding that dementia is second only to cancer as the most feared condition is consistent with similar international studies [11–13, 32, 33]. Notably, the proportions of participants selecting cancer and dementia as most feared in the current Australian study were much more similar (< 5% difference) than found in studies conducted in other countries. This finding may be influenced by a number of possible factors including: improved treatment and survival rates for cancer [34]; cross-cultural differences in the prevalence and care of people with dementia [35]; and/or increases in dementia prevalence [4]. It is also possible that the characteristics of our sample may have contributed to the closer similarity in cancer and dementia fears compared to previous studies. Previous research suggests that there is a significant relationship between poor subjective health and an increased level of dementia worry [22]. Given our sample were recruited from outpatient clinics and were receiving medical care, it is possible that our sample is comprised of a greater proportion of participants with poor subjective health, than in previous studies of the general population.

Despite both cancer and dementia being among the top three leading causes of fatal burden for older Australians [2], a significantly greater proportion of participants aged over 65 years feared dementia the most. Fear for loss of autonomy and cognitive decline associated with dementia may be heightened and more salient for these participants given their stage in life, and they may be more likely to know someone or have known someone living with dementia. Further, people in this age group may also be more likely to have known people who have had positive survival outcomes following a diagnosis of cancer and have witnessed the medical advances in cancer diagnosis and treatment in the last century [36, 37].

Dementia was also the second most feared condition across the younger age categories despite not being a leading cause of fatal burden for these groups in Australia [2]. An international comparative study involving five countries found that people were significantly more likely to indicate a fear of Alzheimer’s disease if they had a personal family experience with condition [38]. Given the rapidly increasing prevalence of dementia, a large proportion of people of all ages have been touched by the condition. A recent study by the authors found that 46% of a sample of Australian outpatients (N = 446) reported knowing someone with dementia [39], with 22% of participants aged below 60 years having a parent or grandparent with the condition (unpublished data).

The top three most important reasons for fearing cancer or dementia, as indicated by participants, were not surprising given general community awareness of the common symptoms and treatment side-effects associated with these conditions. Particularly noteworthy is the finding that only 15.5% reported shortened length of life as a reason for fearing dementia. This may reflect a lack of awareness among the community about the terminal nature of dementia. Dementia is the second leading underlying cause of death in Australia [1] and a recent systematic review and meta-analysis found the average survival time following diagnosis is 4.8 years [40]. An international comparative study found that 61% of participants from the US believed Alzheimer’s is a fatal disease, compared with less than half of their European counterparts [38]. These findings suggest more community education may be needed regarding the impact of dementia on length of life. It is also possible that participants in the current study perceived the impact of dementia on their quality of life to be more important than life expectancy. Further, reasons for fear of dementia specifically could be more deeply explored in research using qualitative methods. Research examining trends in feared conditions over time in Australia may offer further insight into the impact of disease prevalence and public health programs on community fears about dementia and other conditions.

### Implications for public health

Community awareness of dementia is crucial for early detection, addressing stigma, and optimising care [41]. Community fear of dementia, and other conditions, may have some potential benefit in serving as a motivating factor for individuals to engage in preventative health behaviours [42]. The Extended Parallel Processing Model for effective communication of health messages suggests that public health strategies should increase perceived self-efficacy in responding to the threat of disease (danger control response) so as to ensure fear does not lead to maladaptive behaviours, such as denial or avoidance of early signs and symptoms of dementia (fear control response) [43]. Targeted community education programs focusing on effective strategies to reduce the risk of dementia, early symptoms of dementia and when to seek medical advice, as well as information about how to access the range of support services available to assist people to live well with dementia, may be needed. The current findings highlight the need to address fear of disease and reasons for fear in public health messaging about preventable disease, and appropriately harness its potential as a motivating factor to change behaviour.

### Limitations

The implications of study findings are limited by the measurement of the relative fear of conditions rather than the extent that participants fear each condition. Fear of a condition relative to others does not necessarily imply that the level of fear is sufficient to influence psychological wellbeing, health behaviours, or help-seeking. Future studies should consider exploring the absolute level of fear associated with each condition and how fear affects behaviours related to seeking care and support. Findings replicate those found in previous research with community samples, however generalisability of the current findings to the general population is limited due to the convenience sampling method employed within one hospital setting and limited socio-demographic data on participants. Binary gender response options were also a limitation and future research should seek to address this to ensure inclusivity. While data was not collected regarding the current health status of participants, participants were recruited from a broad range of clinics to ensure diversity in the sample. Participants were not recruited from geriatric, memory, or oncology outpatient clinics. It is possible that there may have been some underlying bias in responses as a result of existing health conditions of participants, for example one participant who selected cancer as most feared indicated the reason was due to a current diagnosis of cancer. Future research should explore possible associations between existing health conditions and associated fear of disease.

## Conclusions

Dementia is one of the most feared conditions among Australian health service consumers, second only to cancer. Community education programs to increase perceived self-efficacy of individuals in responding to the threat of dementia are needed to ensure fear of the condition does not lead to negative outcomes, such as denial of early signs and symptoms.

## Data Availability

The datasets used and/or analysed during the current study are available from the corresponding author on reasonable request.
